# Effect of the Refrigeration System on In Vitro Quality and In Vivo Fertility of Goat Buck Sperm

**DOI:** 10.3390/ani10122399

**Published:** 2020-12-15

**Authors:** Eva Mocé, Salvador A. Lozano-Palazón, María del Mar Martínez-Granell, María Lorena Mocé, Ernesto A. Gómez

**Affiliations:** 1 CITA—Instituto Valenciano de Investigaciones Agrarias, Polígono la Esperanza, 100, 12400 Segorbe, Castellón, Spain; martinez_mmagra@gva.es (M.d.M.M.-G.); gomez_ern@gva.es (E.A.G.); 2 ACRIMUR, C/Baron del Solar, 22-A, Edificio II, Entresuelo A, Puerta B, 30520 Jumilla, Murcia, Spain; salva@acrimur.es; 3 Department of Animal Production and Health, Veterinary Public Health and Food Science and Technology (PASAPTA) Facultad de Veterinaria, Universidad Cardenal Herrera-CEU, CEU Universities, C/Tirant lo Blanc, 7, 46113 Alfara del Patriarca, Valencia, Spain; mmoce@uchceu.es

**Keywords:** cooling, chilling, goat buck, semen, liquid-stored semen, refrigerated semen

## Abstract

**Simple Summary:**

Artificial insemination of goats is mostly performed with sperm that has been chilled to 4 °C and stored for up to 12 h. Because the chilling of samples to 4 °C must be done slowly, artificial inseminations have to be performed late in the day, especially in herds located far from artificial insemination centers, which impedes the extensive use of this reproductive technology in goats. In this study, we explored the possibility of chilling sperm doses at a controlled rate (close to −0.18 °C/min) during transportation and compared the quality and fertility of these samples with those chilled in the lab. We observed that the in vitro quality of doses chilled by the prototype procedure developed for transportation was higher than that of samples chilled in the lab, and the fertility of the sperm was similar. Therefore, it is possible to chill doses during transportation without affecting their quality or fertility.

**Abstract:**

Cooling goat sperm insemination doses to 4 °C causes a delay in their delivery. However, chilling these doses during the transportation period could expedite their delivery and the insemination process. In this study, an economical and simple apparatus for chilling goat semen doses in itinere was developed, and the in vitro quality and in vivo fertility of these doses were compared with those chilled by means of a programmable water bath in the laboratory at a rate of −0.18 °C/min. Of the tested prototypes, the one that provided an optimal combination of the chilling rate (average of −0.09 °C/min) and time required to reach 4 °C (3 h 45 min) was selected for further testing. Immediately after chilling and 24 h later, the doses chilled in the prototype were determined to be of higher quality than the samples chilled in the programmable water bath. Finally, the kidding rate was similar between the doses chilled in the programmable water bath (61.7% ± 7.1%) and in the prototype (56.1% ± 5.9%). In conclusion, successful chilling of goat sperm doses during transport is possible, thereby accelerating the delivery of insemination doses.

## 1. Introduction

Artificial insemination (AI) plays an important role in goat breeding programs by facilitating evaluation of the genetic potential of goat bucks in different herds as well as diffusion of the best genetics between herds, reducing sanitary risks [1]. Goat breeding programs undergo rapid advance as the number of inseminated females increases, resulting in higher fertility. However, the use of AI remains low in many dairy goat breeds (about 10% of dairy goats in France and 1% of the total population in Spain [2,3]), which hampers the genetic response of breeding programs.

AI in goats is primarily performed with semen that has been chilled to 4 °C. In Spain, approximately 85% of AIs are performed with sperm at this temperature [4], because its fertility (54–65% [5,6]) is higher than that obtained with frozen semen (35–38% [4]) and similar to that resulting from natural mating in estrous-synchronized goats (74% [7]).

Refrigerated doses for the insemination of goats are stored at 4 °C [5]. At this preservation temperature, protective ingredients (such as egg yolk or skimmed milk) must be added as diluents to prevent cold damage [8]. In addition, chilling must be performed slowly (generally between 1.5 and 4 h [9]) from room temperature to 4 °C to avoid cold shock. However, these protocols delay both the shipment of the AI doses and, consequently, the inseminations.

On the other hand, refrigerated goat buck sperm quickly deteriorates after preparation, which, in turn, jeopardizes its fertilizing ability. For example, the fertility of refrigerated goat buck sperm drastically declines after 12 h of storage [10]. For this reason, most AIs are performed with semen stored for 5–8 h. This limited shelf life interferes with international and even national exchange, depending on the distance between the herds and the AI centers. Moreover, this limitation restricts the advancement of breeding programs since this short lifespan also hinders connections between herds that are far from AI centers [1].

Finally, the combination of the delayed shipment of AI doses due to their slow chilling to 4 °C and the necessity of performing AI with semen stored for less than 12 h is an additional problem that can discourage use of this reproductive technology in this species. However, this problem could be partially alleviated if the doses were chilled during transport (in itinere), which, in turn, would allow dose deliveries to be expedited such that AI could be performed earlier in the workday. To the best of our knowledge, no scientific studies have been conducted for the development of protocols or prototypes to slowly chill sperm doses during their transport to herds. Normally, AI doses are delivered only once they have reached storage temperature, that is, 4 °C. Furthermore, the type of container used for AI in goats (0.25 mL plastic straws) is very sensitive to temperature changes, and for this reason, it is essential to carefully regulate the chilling rate to minimize temperature shock, which is harmful to sperm, affecting its quality.

In this study, we developed an economical and simple system for chilling goat semen doses in itinere. The system was adapted to the commercial coolers that technicians use to transport doses at 4 °C. The in vitro quality and fertilizing ability of doses that were refrigerated in this system were compared with those refrigerated by means of a programmable water bath in the laboratory at a rate of −0.18 °C/min.

## 2. Materials and Methods

### 2.1. Materials and Preparation of Diluents

All chemicals were reagent grade and were purchased from Sigma-Aldrich (Madrid, Spain), except for propidium iodide (PI) and SYBR-14, which were purchased from Invitrogen (Barcelona, Spain). The basic solution to dilute sperm was a Tris–citrate–glucose diluent (TCG; 250 mM of Tris(hydroxymethyl)aminomethane, 83 mM of citric acid anhydrous, and 69 mM of D(+)-glucose; 300 mOsm, pH = 7 [11]). AI doses were prepared with a skimmed milk-based diluent (SM). This diluent was made by adding 0.2 g of D(+)-glucose to 100 mL of skimmed milk (Central Lechera Asturiana, Oviedo, Spain). The diluents TCG and TCG supplemented with bovine serum albumin (3 mg/mL; TCG-BSA) were used for the evaluation of sperm quality, and a sodium chloride solution (0.9% (*w*/*v*)) was used for the determination of sperm concentration.

### 2.2. Animals

This study was performed using adult Murciano-Granadina goat bucks, which were housed in the Centro de Tecnología Animal, Instituto Valenciano de Investigaciones Agrarias (CITA-IVIA; Segorbe, Castellón, Spain). Animals were housed in pens and fed straw and lucerne and 1 kg/day of concentrated feed (17% crude protein, 4.5% crude oils and fat, and 11.6% crude fiber) per male. Fresh water was provided ad libitum. Animal housing and care and protocols for semen collection were approved by the Animal Care and Use Committee of CITA-IVIA and complied with European regulations for the care and use of animals for scientific purposes (RD 53/2013 [12]).

In addition, 522 adult (>18 m) multiparous (≥1 parturition) Murciano-Granadina goats were used for the fertility trial. The females were located in 14 herds from five Spanish provinces (Murcia, Albacete, Avila, Barcelona and Teruel). Females were bred under an intensive production system for dairy-oriented herds. Females were permanently housed in collective yards and managed according to their milk yield and reproductive state. They were provided concentrated feed (adapted to the production level), straw, and lucerne. Fresh water was provided ad libitum. All of the herds belonged to Asociación Española de Criadores de la Cabra Murciano Granadina (ACRIMUR), the official manager of the genetic selection program.

### 2.3. Semen Collection

Ejaculates were collected using an artificial vagina, as described in [13], early in the morning. The ejaculates were placed in a 25 °C water bath until processing. Semen volume was measured by weighing, and the sperm concentration was determined using a spectrophotometer calibrated for goat sperm after diluting the semen 1:400 (*v*/*v*) with a sodium chloride solution.

### 2.4. Preparation of Refrigerated Artificial Insemination Doses

For the preparation of doses, the sperm concentration in each ejaculate was adjusted to 560 × 10^6^ sperm/mL with SM (at 22–25 °C), and an aliquot of 40 µL of each diluted ejaculate was retained for the analyses of sperm quality before chilling (fresh semen). The remaining semen was packed into 0.25 mL plastic straws (IMV Technologies, L’Aigle, France) and sealed with polyvinyl alcohol (PVA, IMV Technologies, L’Aigle, France).

A programmable water bath (Julabo GmbH, Seelbach, Germany) was used following the standard protocol for the refrigeration of straws. In this bath, the straws were chilled from 20 to 4 °C in 90 min at a rate of −0.18 °C/min. The straws were later stored at 4 °C until further use. Immediately after chilling, one straw from each of the ejaculates was taken to analyze sperm quality (refrigerated semen, 0 h).

### 2.5. Sperm Analyses

Motility and sperm plasma membrane integrity (PMI) were evaluated to determine the sperm quality. These analyses were performed according to the protocols described by [14] with modifications. All manipulations were performed at room temperature (22–25 °C). The samples were first diluted to a concentration of 30 × 10^6^ sperm/mL with TCG (D30). The D30 sample was used to perform all dilutions for the analyses of sperm motility and sperm PMI.

Motility characteristics were evaluated for fresh and chilled sperm using a computer-assisted sperm analysis system (CASA; ISAS, version 1.0.17, Proiser, Valencia, Spain) operating at 30 video frames per second (30 Hz); the particle area was set to 15–70 µm, and the search radius was set to 12 µm. A sperm cell was defined as non-motile if the average path velocity (VAP) was lower than 10 µm/s, and it was considered progressively motile when VAP > 75 µm/s and straightness index (STR) ≥ 80%. Sperm motility was assessed at 37 °C using a 10× negative phase contrast objective on a Nikon Eclipse 90i microscope (Nikon Corporation Instruments Company; IZASA, Barcelona, Spain) connected to a computer through a monochrome Basler A312f video camera (Basler Vision Technologies, Proiser, Paterna, Valencia, Spain). For each sample, the sperm concentration was adjusted to 6 × 10^6^ sperm/mL with Tris–BSA, and the samples were incubated at 37 °C for 10 min prior to evaluation.

Evaluations were performed using 5 µL sample aliquots placed on a Makler chamber (Counting Chamber Makler, Sefi-Medical Instruments, Haifa, Israel) that had been pre-warmed to 37 °C on a thermal plate, and data were collected for a minimum of 200 sperm cells from three different fields. Individual sperm tracks were visually assessed to prevent misidentifying debris as tracks. The following variables were measured: percentages of total and progressively motile sperm, average path velocity (VAP; µm/s), curvilinear velocity (VCL; µm/s), straight line velocity (VSL; µm/s), straightness index (STR; %), linearity (LIN; %), wobble (WOB; %), amplitude of the lateral movement of the head (ALH; µm), and beat cross frequency (BCF; Hz).

The percentage of PMI sperm in each sample was determined using flow cytometry. The samples were stained for flow cytometric analysis by transferring 0.1 mL aliquots of the D30 samples into tubes containing 25 µL TCG diluent, 2.5 µL SYBR-14 (10 µM solution in Me_2_SO), and 2.5 µL PI (1.5 mM solution in Milli-Q water). The samples were incubated for 10 min prior to dilution with 0.40 mL of TCG and analyzed using an Epics XL-MCL flow cytometer (Beckman Coulter, IZASA, Barcelona, Spain) that was equipped with standard optics (a 15 mW 488 nm argon ion laser; Cyonics; Coherent, Santa Clara, CA, USA) and EXPO 2000 software (Coulter Corporation, West Lafayette, IN, USA). The green fluorescence of SYBR-14 was detected using a 550 nm longpass (LP) filter combined with a 525 nm (bandwidth 505–545) bandpass (BP) filter (FL1). The red fluorescence of PI was detected using a 645 nm LP filter combined with a 620 nm (bandwidth 605–635) BP filter (FL3). The photomultiplier (PMT) value of the detector was set at 650 V in FL1 and 691 V in FL3. FL1–FL3 compensation was 41.4%, and FL3–FL1 compensation was 5.5%. At least 10,000 events per sample were analyzed. Using this protocol, all cells stained with SYBR-14, are distinguished from non-DNA-containing events, while only non-viable cells are stained with PI. Non-DNA-containing events (SYBR-14- and PI-negative) were not included in the calculations to prevent overestimation of the proportion of PMI (PI-negative) sperm [15,16]. The percentages of PMI sperm (SYBR-14-positive and PI-negative) were included in the analysis.

### 2.6. Estrous Synchronization and Artificial Insemination

Estrous was synchronized using the short protocol described by [17] with modifications. Only females in good body condition with high milk yield and normal transabdominal ultrasound images of the uterus (without signs of reproductive pathologies or pregnancy) were selected for the trial. On day 0, intravaginal pessaries with 30 mg of flugestone acetate (FGA, SINCROPART^®^ 30MG CEVA, Salud Animal, Barcelona, Spain) were introduced using an applicator lubricated with a gel (BOVIVET Gel 1000 mL, Kruuse, Langeskov, Denmark). The applicator was disinfected with a quaternary ammonium disinfectant (CATIGENE PLUS, Quimicamp Higiene S.L., Valencia, Spain) between use on different females. When the pessaries were inserted, each female received an intramuscular (IM) injection of 2.5 mg prostaglandin F2α (Enzaprost^®^ T, CEVA, Salud Animal, Barcelona, Spain). The pessaries were removed on day 6, and each female received an IM injection of 250 IU (for the AIs performed in autumn or winter) or 300 IU (for the AIs performed during spring or summer) of PMSG (SINCROPART^®^ PMSG 6000 IU, CEVA, Salud Animal, Barcelona, Spain).

AIs were performed on day 8, between 45 and 48 h after PMSG injection. Goats were inseminated via the cervix using a speculum that was lubricated with gel (BOVIVET Gel 1000 mL, Kruuse, Langeskov, Denmark) and equipped with an attached light source and an ovine–caprine AI catheter (IMV Technologies, L’Aigle, France). Cervical mucus, if present, was carefully removed before depositing the semen since its bacterial and inflammatory products affect sperm viability [18]. Semen was carefully deposited as deep as possible in the cervix while preventing harm to the cervix epithelia and semen reflux. Each female was inseminated with the contents of one straw (140 × 10^6^ total sperm). The speculum was disinfected between females with quaternary ammonium disinfectant (CATIGENE PLUS, Quimicamp Higiene S.L., Valencia, Spain).

### 2.7. Experimental Design

Three experiments were conducted. In the first experiment, several variants of the prototype for the refrigeration of doses during transport were tested to determine which one produced a chilling rate closest to −0.18 °C/min (the chilling rate of the programmable water bath used in the laboratory). In the second experiment, the quality was compared between doses that were refrigerated in the programmable water bath and those cooled in the prototype. In the third experiment, the in vivo fertilizing ability was determined.

#### 2.7.1. Experiment 1: Development of a Simple Prototype for Refrigerating Sperm Doses during Transport

The chilling rates were first measured using several prototypes that are compatible with the portable compressor coolers used by technicians who perform AIs. The objective was to develop a simple system with a chilling rate that was as close as possible to the chilling rate of the programmable water bath (−0.18 °C/min). The prototype consisted of a glass jar (17 cm height × 5.5 cm diameter) in which goblets containing the straws and a temperature data logger (Temperature Data Logger RC-5; Elitech, London, UK) were located. The temperature data logger recorded the temperature every minute. The glass jar was then submerged in 750 mL of water at room temperature in a plastic container with dimensions of 18 cm × 10 cm × 10 cm (height × length × width). The water surrounded the glass jar to a height above the level of the straws and without coming into direct contact.

To obtain different chilling temperatures, a variable number of ice blocks (200 g each and 16 cm × 10.5 cm × 1 cm (height × length × width)) were fixed to 0, 1, 2, or 3 of the surrounding plastic container walls with rubber bands. The ice blocks had been prepared by freezing at −25 °C in a conventional freezer. Then, the entire system was placed into a portable compressor cooler at 4 °C. In the preliminary experiment, a prototype with four ice blocks (surrounding all four walls of the plastic recipient) was tested. However, the ice blocks were not in contact with the total wall surface because the width of the ice blocks was slightly greater than the surface of the plastic recipient walls. For this reason, this variant was not considered in this study.

The temperature was monitored, and the data from the logger were downloaded to an Excel file with the program URC-5 (Elitech, London, UK). Data were plotted to generate temperature drop curves. In addition, the chilling rate for each minute was calculated by subtracting the temperature measured at each minute from the temperature recorded at the previous minute. The chilling rates obtained in the first 100 min of cooling for each system were also plotted in charts. The rate for each minute was calculated as the average of the values obtained for the minute in question and those at the previous and subsequent minutes. Of all tested variants, the one that provided the best combination of the chilling rate and time required to reach 4 °C, i.e., to match the use of the programmable water bath, was selected for the remaining experiments.

#### 2.7.2. Experiment 2: Comparison of In Vitro Sperm Quality of Doses Chilled to 4 °C Using either the Programmable Water Bath (PWB) or Selected Prototype (Portable)

The quality of the sperm in samples chilled using the programmable water bath (from 20 to 4 °C in 90 min) was compared with those cooled in the variant with 3 ice blocks (sperm doses reached 4 °C in 3 h 45 min). For this experiment, 24 ejaculates from 12 Murciano-Granadina male bucks (2 ejaculates/male) were used. Ejaculates were processed according to the previously described protocol, and after they had been packaged into the straws, half of the samples were chilled in the programmable water bath, while the other half were cooled in the prototype developed for the portable compressor cooler. To prevent the temperature from decreasing below 4 °C, the ice blocks were removed from the system at 3 h 45 min. The quality of the samples (sperm motility and sperm PMI) was evaluated immediately after the samples reached 4 °C (0 h) and 24 h later according to the previously described protocols.

To determine the average quality of fresh and refrigerated semen, a descriptive analysis of the data was first performed. Then, paired samples were compared using R [19] to increase the statistical power of the tests. The planned comparisons were Fresh vs. PWB 0 h and Portable 0 h; PWB 0 h vs. Portable 0 h; and PWB 24 h vs. Portable 24 h. If the differences for matched pairs followed a normal probability distribution, a t-test was performed. If not, a non-parametric test was used (Mann–Whitney–Wilcoxon test).

#### 2.7.3. Experiment 3: Fertilizing Ability of the Doses Refrigerated in the Programmable Water Bath vs. during Transport

In the third experiment, an in vivo fertility trial was conducted to determine the fertilizing ability of the doses refrigerated during transport vs. the programmable water bath. Goats (n = 522) were inseminated, and the number of inseminations varied among herds (from 17 to 62 inseminations). In five herds, the doses were refrigerated in the programmable water bath (for inseminating 235 goats), and in the other nine, the doses were chilled during transport (for inseminating 287 goats). The Murciano-Granadina female goats belonged to commercial herds that were located from 87 km (50 min of traveling) to 489 km (5 h 17 min of traveling) from the AI center. Estrous synchronization and AIs were performed according to the previously described protocol. Kidding goats were recorded at parturition.

Sixty-four ejaculates from thirteen males were used to produce the doses; there were variations in the number of doses inseminated per male and herd (between 1 and 23) and the number of herds where the males were used, depending on the insemination schedule of the breeding program. The doses were prepared according to the previously described protocol, and they were chilled to 4 °C in the programmable water bath (and were delivered once they had reached 4 °C) or in itinere in the portable compressor cooler containing the prototype with three ice blocks (whereby the doses reached 4 °C in 3 h 45 min, after which the ice blocks were removed to prevent further decreases in the temperature).

In Experiment 3, data were analyzed using SAS/STAT^®^ software [20], accounting for the binomial nature of fertility (expressed as kidding success after insemination). A generalized linear mixed model (mixed logistic regression; GLIMMIX procedure) with one fixed effect (cooling system, two levels) and two random effects (herd and buck) was performed, and the expansion of the restricted pseudo-likelihood with Taylor series was used as the estimation method [21]. Estimates of least-square means of the predicted probabilities (and their standard errors) are reported on the logit scale and on the real scale of the data (inverting the logit function) to provide a comprehensive understanding of the results. Additionally, estimates of the covariance parameters (and asymptotic standard errors) of random effects are shown.

## 3. Results

**Figure 1:**
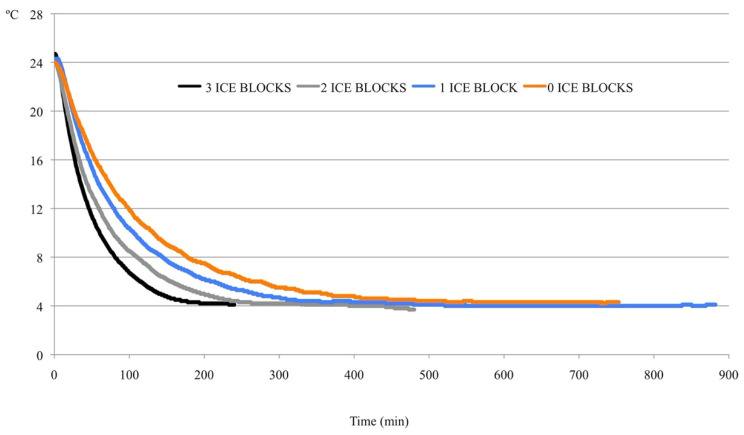
Temperature drop in different variants of the prototype adapted to the portable compressor cooler according to the number of ice blocks. The *Y*-axis represents the temperature (°C), and the *X*-axis represents time (min).

The temperature decrease in each of the tested prototypes is shown in Figure 1.

**Figure 2:**
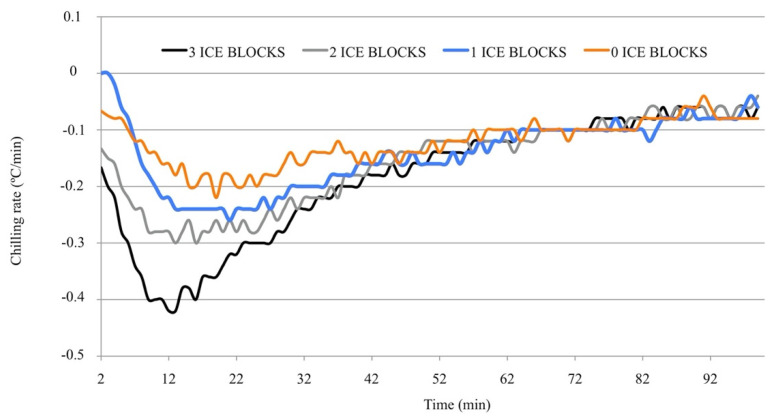
Chilling rates in different variants of the prototype adapted to the portable compressor cooler during the first 100 min of cooling. The *Y*-axis represents the chilling rate (°C/min), and the *X*-axis represents time (min).

The chilling rates of the different systems for the first 100 min are plotted in Figure 2. As expected, the chilling rate in the prototype decreased as the number of ice blocks decreased. Irrespective of the number of ice blocks used, the chilling rate seldom surpassed −0.4 °C/min. The chilling rate with one or no ice blocks never exceeded −0.3 °C/min. When three and two ice blocks were used, chilling rates of −0.4 and −0.5 °C/min were reached in only 10 and 2 min of the total period necessary to reach 4 °C (more than 200 min in all of the prototypes). After 60 min, the chilling rates were very similar for all variants of the prototype, irrespective of the number of ice blocks.

It took 3 h 45 min, 6 h 33 min, 8 h 42 min, and 12 h 10 min to reach 4 °C with three, two, one, and zero ice blocks, respectively. After these time points, it was necessary to remove the ice blocks from the cooler to prevent further decreases in the temperature. The variant with three ice blocks provided an adequate combination of the chilling rate and the time necessary to reach the storage temperature (4 °C). Therefore, this variant was used in subsequent experiments. When fewer ice blocks were used, the slope in the first part of the curve (up to 60 min) was smooth, but more time was required to reach 4 °C, because the chilling rate was very slow. For example, it took 31 min to reach 15 °C when three ice blocks were used, whereas 38, 52, and 64 min were required when two, one, and zero ice blocks were used, respectively. In addition, with three ice blocks, the doses reached 10 °C after 61 min, which increased to 78, 106, and 132 min for two, one, and zero ice blocks, respectively.

The results from the second experiment are summarized in Table 1 (description) and 2 (analysis). Only 4 out of 44 comparisons did not follow normal distributions and were analyzed with non-parametric tests (once for the variables VAP and BCF and twice for the variable WOB). Therefore, the t-test was used for 90.9% of the comparisons. Compared with fresh semen, the doses refrigerated in the programmable water bath had higher values (*p* < 0.05) of VCL, VSL, VAP, LIN, and WOB and lower values of PMI sperm (*p* < 0.05) immediately after chilling (0 h) (Table 2). However, more similarities were observed between the quality of the doses chilled in the prototype with three ice blocks developed for the portable compressor cooler and that of fresh semen. Indeed, significant differences (*p* < 0.05) between them were only observed for VCL, VAP, and ALH, which were higher for the doses chilled in the portable compressor cooler than for fresh semen.

When the sperm quality was compared immediately after chilling (0 h) in the programmable water bath and in the portable compressor cooler, several differences were observed. Samples chilled in the programmable water bath had lower values (*p* < 0.05) of TMS, VCL, VAP, STR, ALH, and PMI and higher values (*p* < 0.05) of LIN and WOB than samples chilled in the portable compressor cooler. Moreover, most of these differences were also observed 24 h after chilling, except for VAP, LIN, STR, and WOB, which were similar between the two refrigeration systems, and BCF, which was lower (*p* < 0.05) for the doses chilled in the programmable water bath than for those chilled in the portable compressor cooler.

In the third experiment, the fresh ejaculates contained an average of 2751 × 10^6^ sperm/mL and 77.4% total motile sperm, 62% progressively motile sperm and 66.3% PMI sperm (data not included in the tables). The quality of chilled doses did not differ between refrigeration systems and had an average of 75.1% total motile sperm, 60.1% progressively motile sperm and 54.7% PMI sperm. Therefore, after refrigeration, the doses retained 97% of the total motile sperm, 97% of the progressively motile sperm, and 82% of the PMI sperm that were present before refrigeration (data not included in tables). Taking into account the percentage of total motile sperm of the chilled doses, each female was inseminated with an average of 105 × 10^6^ total motile sperm. The average kidding rate was 56.3% and was similar for goats inseminated with doses chilled for all of the different cooling systems (programmable water bath or prototype with three ice blocks) (Table 3).

## 4. Discussion

Skimmed milk diluents are widely used for the preparation of AI doses in goats [5]. Refrigerated AI doses for goats can be preserved between 2 and 15 °C, but 4 °C is recommended when skimmed milk diluents are used [22]. To minimize cold damage in AI doses when reducing the temperature to 4 °C, cooling must be performed slowly. One of the consequences of this slow cooling rate is the delayed delivery of insemination doses from the AI center to technicians. Chilling the doses during transport would expedite their delivery. An economical and simple prototype was developed in the first experiment, and a prevailing cooling rate of ≤0.2 °C/min was obtained in all variants, which is very close to the cooling rate achieved with the programmable water bath (−0.18 °C/min). As expected, the cooling rate in the prototype increased as the number of ice blocks increased.

Previous studies have demonstrated that fast cooling rates (>0.55 °C/min) are detrimental to the in vitro quality of goat buck sperm [23,24,25]. However, the effects of intermediate cooling rates on sperm quality remain unknown. In addition, some authors have observed that goat buck sperm quality was susceptible to rapid cooling (0.6 °C/min) from 30 to 15 °C when it was diluted with an extender that lacked cold protective ingredients (such as egg yolk or skimmed milk) [24]. The cooling rates in the tested variants seldom exceeded 0.4 °C/min and never reached 0.6 °C/min. For this reason, it is unlikely that the cooling rates reached in the first section of the curve are detrimental to the sperm.

The variant with three ice blocks provided the most suitable combination of the cooling rate and the time necessary to reach the storage temperature (4 °C), so this system was chosen for subsequent experiments. The effect of slower cooling rates on in vitro sperm quality has never been investigated, and the objective was to obtain a cooling rate that was as close as possible to the recommended rate of 0.18 °C/min. Scientific evidence suggests that a temperature of 4 °C is more appropriate for storing goat buck sperm than higher temperatures (15 °C [22]) when skimmed milk diluents are used. Furthermore, there is an increasing emphasis on avoiding the prophylactic use of antibiotics, including those in semen extenders. In the absence of antibiotics, there is a risk of excessive microbial growth if the doses are cooled extremely slowly since it takes longer than 6 h 30 min to reach 4 °C. It has previously been demonstrated that a temperature of 4 °C has a bacteriostatic effect for at least 24 h in extended goat buck sperm that has been contaminated with bacteria [26]. Therefore, prolonging the time to reach a storage temperature of 4 °C is not justifiable considering either in vitro sperm quality [22] or microbiological contamination [26].

Sperm doses chilled in the prototype adapted to the portable compressor coolers were of higher quality than those cooled in the programmable water bath, as demonstrated by the percentages of total motile and plasma membrane intact sperm observed at 0 and 24 h. Indeed, the quality of samples that were chilled in the prototype was extremely close to that of fresh semen. Thus, the doses refrigerated in the programmable water bath retained 91% of the total motile sperm, 98% of the progressively motile sperm, and 76% of the PMI sperm in the samples before cooling; for the samples chilled in the prototype, these retention rates were 100% of total motile sperm, 100% of progressively motile sperm and 98% of PMI sperm. In addition, the doses chilled in the prototype exhibited higher velocities (VCL and VAP) and less linear trajectories (lower LIN and STR) than the samples chilled in the programmable water bath. In a previous study, these characteristics (high speed and non-linear spermatozoa) were associated with high-quality spermatozoa [27].

The reason for this small difference in sperm quality between the two systems is unknown, although there are some possible explanations. The average chilling rate in the prototype (−0.09 °C/min) might be more important than the point-by-point rates in the curve, even for a non-linear decrease in the temperature obtained with the prototype. This cooling rate is slower (half the chilling rate) than that obtained in the programmable water bath and could have an effect on sperm quality. These results corroborate those from previous studies in goats [23,24,25], although in those studies, the observed differences between doses that were refrigerated with slow and fast freezing rates were more pronounced for all of the evaluated parameters. These differences in results between studies could be due to the breed (Boer, Blanca Celtiberica, or Murciano-Granadina), diluent (skimmed milk, egg yolk, or mZAP) or, more likely, the rate of cooling that was considered fast (these cooling rates were faster than that in the programmable water bath in our study).

Furthermore, goat buck sperm might benefit from non-linear chilling rates. This means that it might be better to chill the sperm at different rates depending on the time interval, with faster rates (even higher than −0.18 °C/min) used until reaching an intermediate temperature (such as 15 or 10 °C) and slower rates (as low as −0.1 °C/min) from the intermediate temperature to 4 °C. However, studies to probe this theory have never been conducted, and we cannot categorically assert that this is the reason that doses chilled in the prototype were of higher quality than those chilled in the programmable water bath. Nevertheless, it remains to be determined whether the differences between doses chilled with the two systems are large enough to have a physiological impact. After 24 h of storage at 4 °C, some of the differences between the chilling systems remained, although differences in the indexes LIN, STR, and WOB disappeared. Remarkably, at 24 h of storage at 4 °C, there was an increase in the non-linear or circular trajectories (characterized by lower VSL, LIN, and STR), and higher ALH values were observed in both chilling systems together with an expected decrease in the percentages of total motile, progressively motile, and PMI sperm. These trajectories are followed by more erratic sperm and are indicative of degenerative processes or compromised metabolism [28].

The average kidding rate in our study (56.3%) is similar to the fertility rate previously reported for this breed (55–65% [4]) and other breeds (between 54.3% and 64.8% [5,6]). In addition, fertility was not affected by the cooling system. This result was expected since the sperm quality indicators obtained after chilling in the two systems studied in this work were similar. Furthermore, the number of motile sperm used for insemination (105 × 10^6^ motile sperm/AI on average) was higher than the numbers necessary to reach maximum fertility in doe goats that had undergone cervical insemination with fresh diluted (60 × 10^6^ motile sperm/AI [29]) and frozen–thawed semen (between 60 × 10^6^ and 80 × 10^6^ motile sperm/AI [29,30]). It is well known that fertility usually increases to a maximum as the sperm numbers in the AI doses increase and that the minimum numbers of sperm to reach maximum fertility differ between individual males (in bulls [31]). However, the AI doses are often increased to provide greater confidence that sperm numbers are well in excess of minimum thresholds and that the fertility will not be affected [31]. As a result, the “real” fertility of the doses is masked, which likely occurred in our study.

Therefore, it is possible to cool goat sperm doses during transport because there is no decline in either the sperm quality or the sperm fertilizing ability. Moreover, at least 90 min is saved, allowing AIs to be scheduled earlier in the workday, which is both convenient and increases the time window of potential use before there is a decline in dose quality due to long-term storage.

## 5. Conclusions

In conclusion, sperm doses cooled in the prototype with three ice blocks adapted to portable compressor coolers were of higher quality than those cooled in the programmable water bath. In addition, the fertility of doses cooled in itinere was similar to that obtained with doses that were refrigerated in the programmable water bath and to that reported by other authors for refrigerated goat buck sperm. Therefore, this prototype allows goat sperm doses to be cooled during transport, allowing its delivery from AI centers to be brought forward by at least 90 min (or more, depending on the time necessary to verify the quality of the sperm doses once cooling is completed), and technicians can perform AIs earlier in the workday. Finally, this prototype can be easily applied to cool sperm of other species.

## Figures and Tables

**Table 1:** Descriptive analyses for the variables of goat buck sperm quality for fresh samples and samples chilled to 4 °C in the programmable water bath (PWB) or in the prototype with three ice blocks developed for the portable compressor cooler (Portable) immediately after reaching 4 °C (0 h) and 24 h later (24 h).

Variable	Fresh Semen		PWB 0 h		Portable 0 h		PWB 24 h		Portable 24 h	
	Mean	SD	Mean	SD	Mean	SD	Mean	SD	Mean	SD
TMS (%)	76.2	9.2	69.0	17.5	76.7	10.8	56.5	22.6	63.6	23.8
PMS (%)	47.8	10.0	46.9	17.0	48.1	17.0	26.8	19.0	29.7	16.0
VCL (µm/s)	134.3	22.8	141.7	20.8	151.4	22.4	139.6	35.3	153.3	23.2
VSL (µm/s)	90.5	20.8	97.0	17.6	96.3	20.9	67.3	22.4	72.5	15.1
VAP (µm/s)	111.7	23.6	119.6	20.1	124.5	21.6	99.6	28.5	107.1	17.5
LIN (%)	66.3	10.2	69.9	10.9	65.6	13.5	49.6	14.3	50.8	11.5
STR (%)	78.0	7.5	79.6	7.7	75.5	11.3	65.1	12.5	66.9	9.2
WOB (%)	82.0	7.5	84.6	7.4	82.6	8.2	71.2	9.9	71.7	8.5
ALH (µm)	2.9	0.6	2.8	0.7	3.2	0.8	3.8	1.0	4.2	0.9
BCF (Hz)	12.0	1.6	11.8	1.5	11.9	0.9	11.4	2.1	12.2	1.0
PMI (%)	67.7	12.1	51.2	17.6	66.3	13.5	49.3	18.7	60.4	20.6

Results are expressed as the mean and the standard deviation (SD). TMS: total motile sperm; PMS: progressively motile sperm; VCL: curvilinear velocity; VSL: straight line velocity; VAP: average path velocity; LIN: linearity index; STR: straightness index; WOB: wobble; ALH: amplitude of the lateral movement of the head; BCF: beat cross frequency; PMI: plasma membrane intact sperm.

**Table 2:** Comparisons of differences in the quality of the fresh semen and that of the doses chilled in the programmable water bath (PWB) or in the prototype with three ice blocks developed for the portable compressor cooler (Portable) immediately after reaching 4 °C (0 h) and 24 h later (24 h).

	Comparisons			
Variables	PWB 0 h vs. Fresh	Portable 0 h vs. Fresh	PWB 0 h vs. Portable 0 h	PWB 24 h vs. Portable 24 h
TMS (%)	−7.27	0.50	−7.77 *	−7.14 *
PMS (%)	−0.95	−0.32	−1.27	−2.86
VCL (µm/s)	7.34 *	17.01 *	−9.67 *	−8.24 *
VSL (µm/s)	6.46 *	5.80	0.67	−2.01
VAP (µm/s)	7.90 *	12.8 *	−4.93 *	−3.34 ^1^
LIN (%)	3.60 *	−0.71	4.30 *	0.44
STR (%)	1.50	−2.50	4.10 *	−0.17
WOB (%)	2.6 *^,1^	0.70	1.9 *^,1^	0.60
ALH (µm)	−0.04	0.31 *	−0.35 *	−0.30 *
BCF (Hz)	−0.13 ^1^	−0.08	−0.05	−0.48 *
PMI (%)	−16.47 *	−1.36	−15.12 *	−11.04 *

Results are expressed as the value of the difference between the compared treatments. ^1^ Indicates that results were analyzed with a non-parametric test. * Indicates that the average difference is different from zero (*p* < 0.05); TMS: total motile sperm; PMS: progressively motile sperm; VCL: curvilinear velocity; VSL: straight line velocity; VAP: average path velocity; LIN: linearity index; STR: straightness index; WOB: wobble; ALH: amplitude of the lateral movement of the head; BCF: beat cross frequency; PMI: plasma membrane intact sperm.

**Table 3:** Kidding rates of the AI doses chilled using the programmable water bath (PWB; N = 235 inseminated goats) or the prototype with 3 ice blocks developed for the portable compressor cooler (Portable; N = 287 inseminated goats).

Statistical Outcomes	Kidding Rate	
	PWB	Portable
Probabilities ^1^	0.48 ± 0.30	0.25 ± 0.24
Data scale (%) ^2^	61.7 ± 7.1	56.1 ± 5.9
Cov. Parameters ^3^		
Buck	0.072 ± 0.10	
Herd	0.30 ± 0.18	
Events/trials	294/522 (56.3%)	

^1^ Least-square means (±standard error) of predicted probabilities on the logit scale. ^2^ Estimates of the kidding rates (±standard error, computed by the delta method) on the real scale by group. ^3^ Estimates of covariance parameters (±asymptotic standard error) of random effects.
